# Supplementary material for: YAP1/TAZ-TEAD transcriptional networks maintain skin homeostasis by regulating cell proliferation and limiting KLF4 activity
Source: Nat Commun. 2020 Mar 19;11:1472. doi: 10.1038/s41467-020-15301-0 (PMC7081327; doi:10.1038/s41467-020-15301-0)
Supplement: Supplementary file 4 — Supplementary Information [file 41467_2020_15301_MOESM4_ESM.pdf]

Supplementary Information for:

YAP1/TAZ-TEAD transcriptional networks maintain skin homeostasis by regulating cell proliferation and limiting KLF4 activity

Yuan et al.

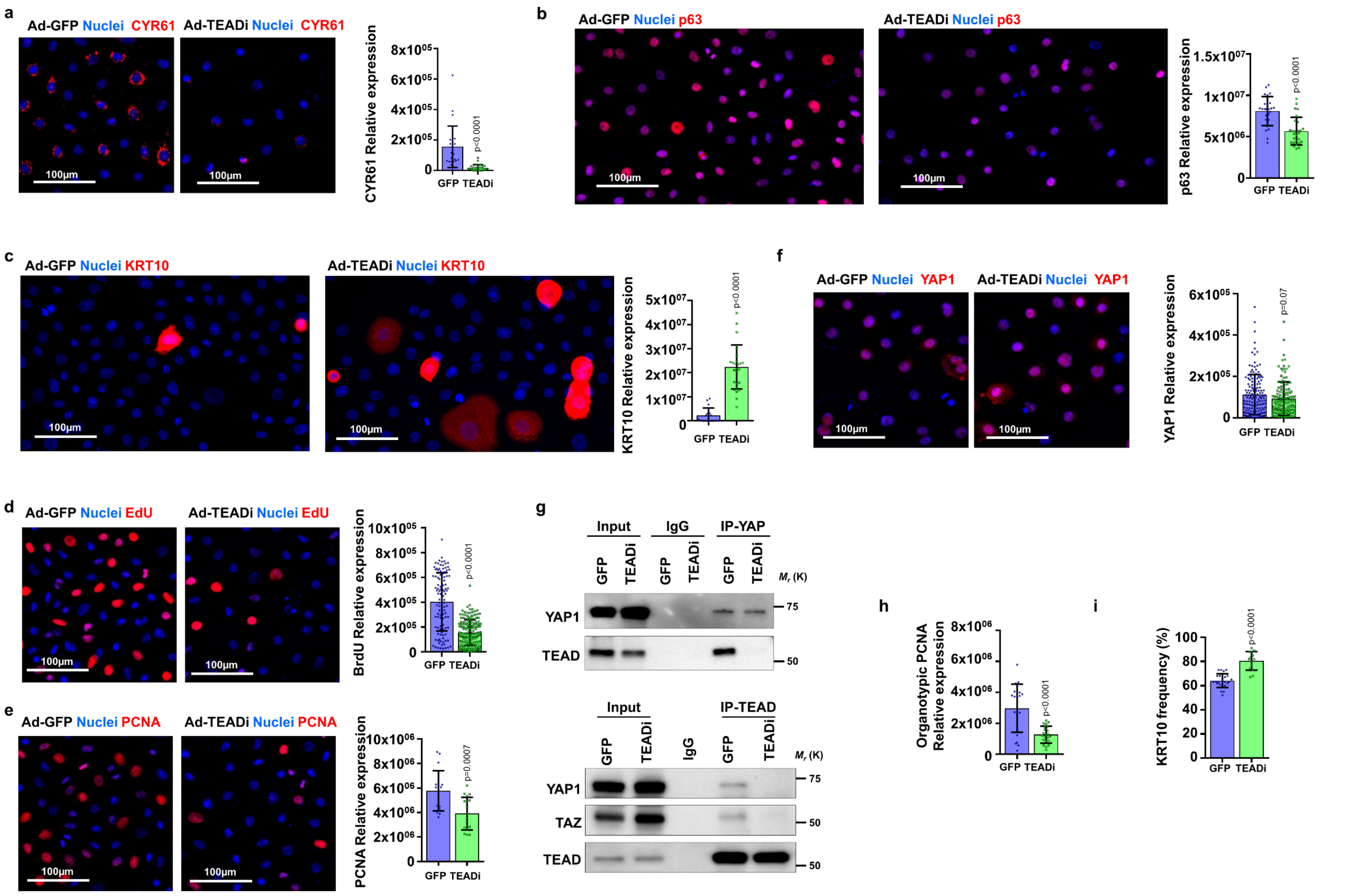

Supplementary Figure 1: **TEAD-inhibition triggers rapid keratinocyte cell cycle arrest and differentiation. (a to f)** IF staining showing the expression and quantification of the indicated markers in N/TERT2G cells transduced with GFP (Ad-GFP, control) or TEADi (Ad-TEADi) for 48 hs. **(g)** Coimmunoprecipitation experiments in N/TERT2G keratinocytes transduced with GFP (Ad-GFP, control) or TEADi (Ad-TEADi) for 48 hs. **(h and i)** Quantification of PCNA and KRT10 expression in control (GFP) or TEADi organotypic cultures. In (a) n=27 fields, (b) n=27 fields, (c) GFP n=17 and TEADi n=27 fields, (d) GFP n=115 and TEADi n=173 fields, (e) n= 18 fields, (f) GFP n=152 and TEADi n=135 fields, (h) n=22, (i) GFP n=24 and TEADi n=14 fields from 3 biological replicates. Mean ± SD is shown; two-tailed unpaired t test. Source data are provided as a Source Data file.

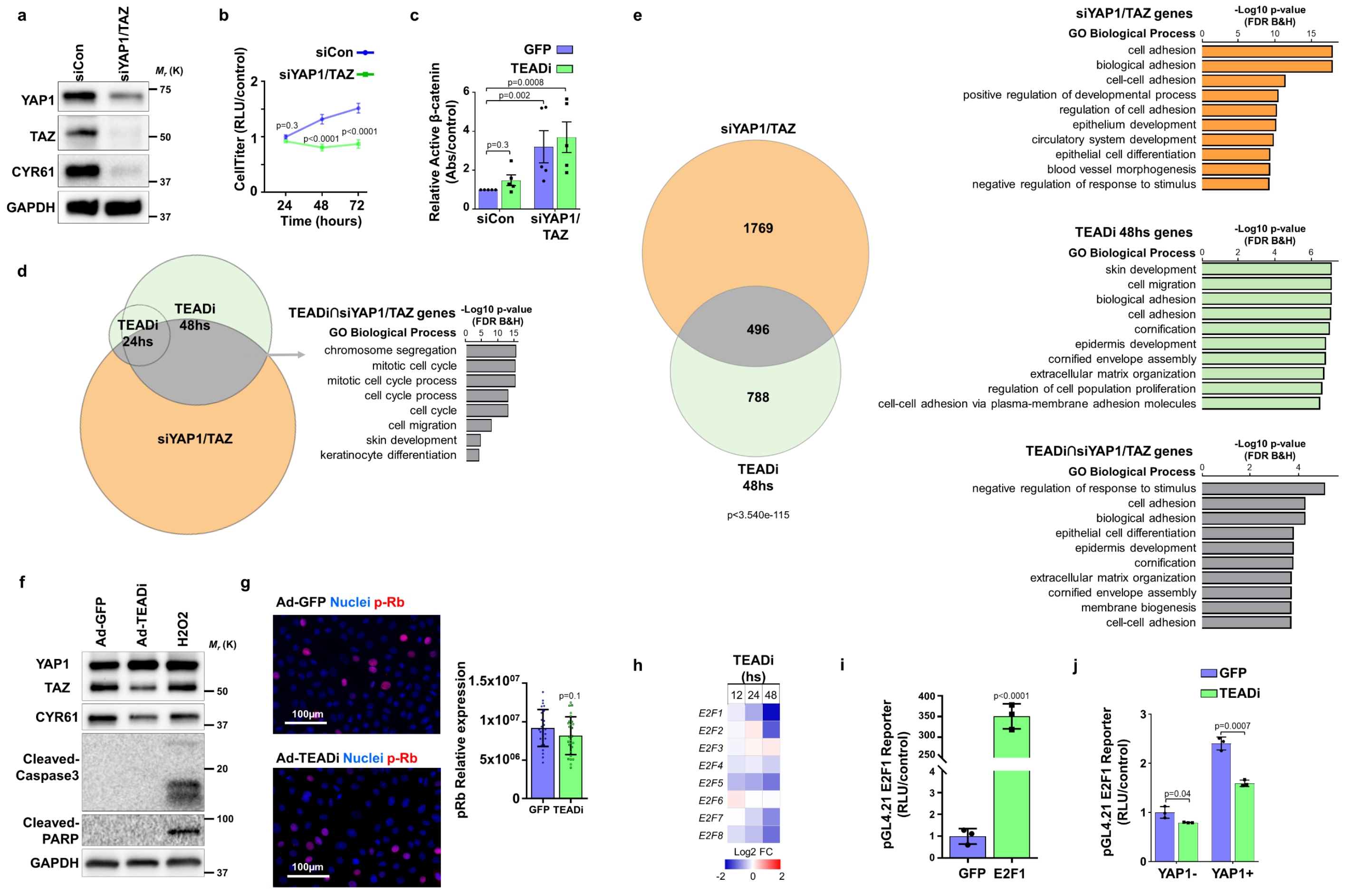

Supplementary Figure 2: **TEAD transcriptional networks in keratinocytes regulate cell cycle entry. (a)** Western blot analysis of the expression of YAP1, TAZ and CYR61 in N/TERT2G cells transduced with pooled siRNAs for 48hs. **(b)** Luminescence cell proliferation assay in N/TERT2G cells transduced with control siRNA (siCon) or siRNA targeting YAP1 and TAZ (siYAP1/TAZ). **(c)** Quantification of the levels of active non-phospho active β-Catenin (Ser45) from independent experiments as shown in Fig. 2g. **(d)** Venn diagram showing the overlap between differentially regulated genes ($q<0.05$, $|FC|\geq1.5$) by TEADi and siYAP1/TAZ in N/TERT2G keratinocytes and graph indicating selected GO biological process terms from genes in the highlighted gray intersection (∩). **(e)** Venn diagram showing the overlap between differentially regulated genes at higher stringency levels ($q<0.01$, $|FC|\geq2$) in the TEADi and siYAP1/TAZ datasets and graphs indicating the top ten GO biological process terms enriched in the genes present in each entire dataset or just the intersection (∩). **(f)** Western blot analysis of the expression of the indicated markers in N/TERT2G cells transduced GFP (Ad-GFP, control) or TEADi (Ad-TEADi) for 48hs or treated with hydrogen peroxide (H2O2) to trigger apoptosis as a positive control. **(g)** IF staining showing the expression and quantification of phosphorylated Rb protein (p-Rb) in N/TERT2G cells transduced with Ad-GFP (control) or Ad-TEADi for 48hs. **(h)** Graph indicating the fold change (Log2 FC) of E2F family genes in N/TERT2G cells transduced with TEADi compared with GFP expressing cells. **(i and j)** Transcriptional activity of a luciferase reporter containing 1.7kb of the proximal promoter of human *E2F1* gene in HEK293 cells transfected with the indicated transcripts. In (b) n=4 biological replicates; (c) n=5 biological replicates; (g) n=27 fields from 3 biological replicates; (i) and (j) n= 3 biological replicates. Mean ± SD is shown in (b), (g), (i) and (j); mean ± SEM is shown in (c); (b) and (c) two-way ANOVA with Sidak's multiple comparison test; (g), (i) and (j) two-tailed unpaired t test. In (e) p indicates the p-value of the overlap in the Venn diagram, Fisher's exact test. Source data are provided as a Source Data file.

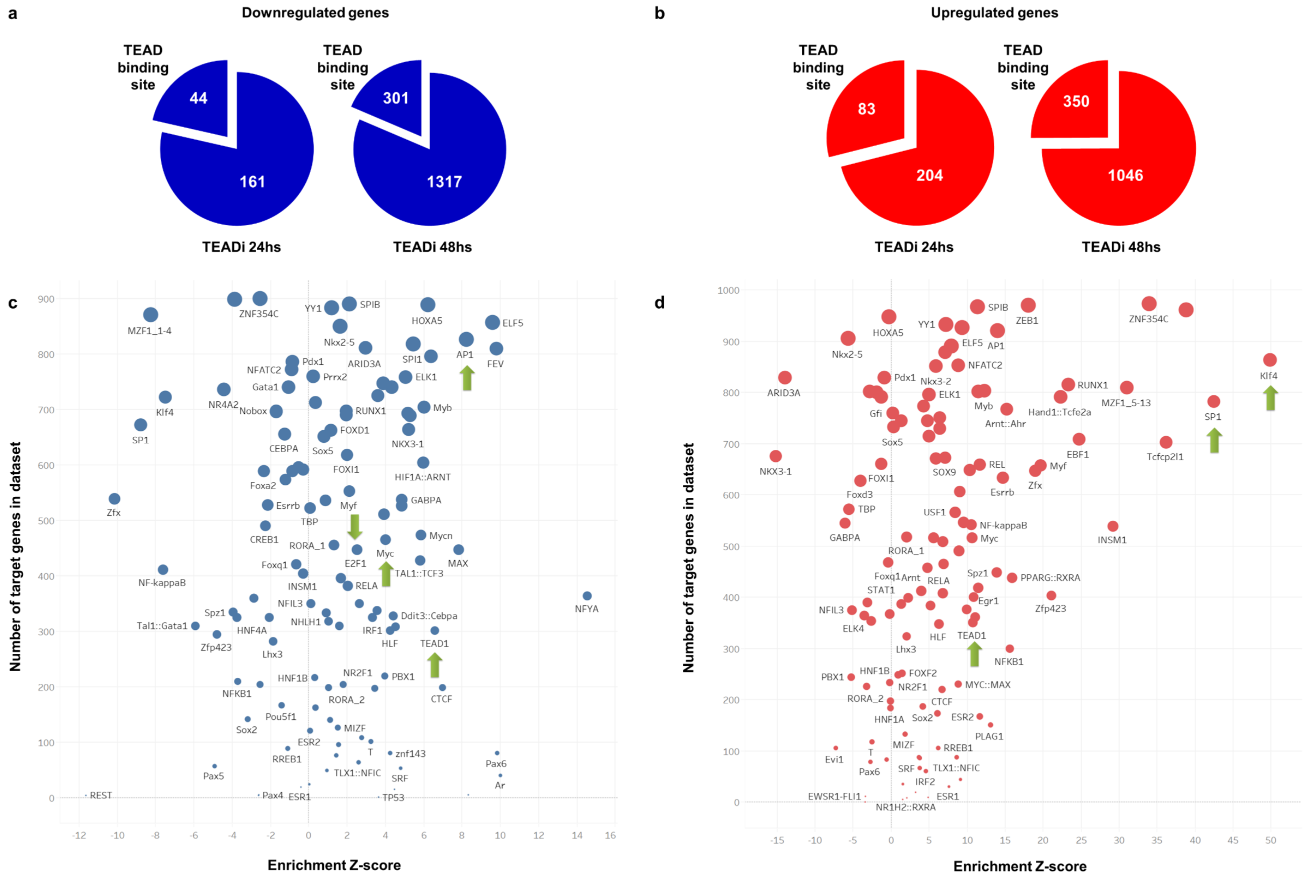

Supplementary Figure 3: Transcriptional networks regulated by TEAD in keratinocytes. (a and b) Proportion of significantly differentially regulated genes in N/TERT2G cells by TEADi at 24 and 48 hs with predicted TEAD binding sites in promoter regions. **(c and d)** Transcription factors found in the predicted transcription factor binding site enrichment analysis of TEADi upregulated and downregulated gene sets at 48hs. Size of circle is correlated with the number of target genes in dataset for that transcription factor. Transcription binding site analysis was performed with oPOSSUM [1]. Source data are provided as a Source Data file.

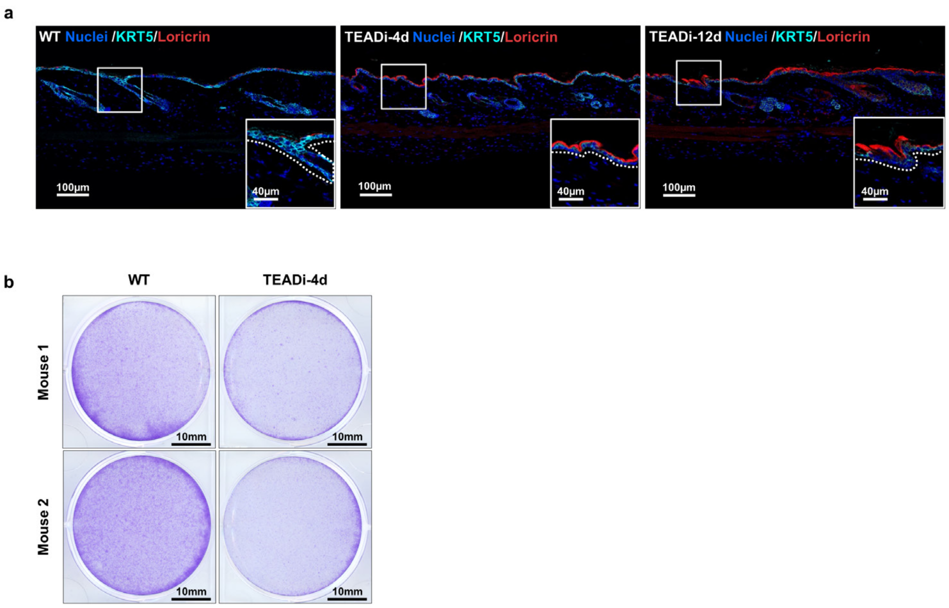

Supplementary Figure 4: TEAD blockage in the mouse skin induces keratinocyte differentiation and progenitor cell depletion. (a) Representative IF pictures of the differentiation marker loricrin and the basal marker KRT5 in the skin of WT or TEADi mice induced for 4 or 12 days. Inserts show magnification of highlighted area and location of the basal membrane is indicated with a white dotted line. **(b)** Representative pictures of wells from clonogenic assays of keratinocytes isolated from 2 littermate controls (WT) and 2 TEADi mice 4 days after induction.

Supplementary reference

1. Kwon AT, Arenillas DJ, Worsley Hunt R, Wasserman WW. oPOSSUM-3: advanced analysis of regulatory motif over-representation across genes or ChIP-Seq datasets. *G3 (Bethesda)* **2**, 987-1002 (2012).
