# Supplementary material for: YAP1/TAZ-TEAD transcriptional networks maintain skin homeostasis by regulating cell proliferation and limiting KLF4 activity
Source: Nat Commun. 2020 Mar 19;11:1472. doi: 10.1038/s41467-020-15301-0 (PMC7081327; doi:10.1038/s41467-020-15301-0)
Supplement: Supplementary file 5 — Reporting Summary [file 41467_2020_15301_MOESM5_ESM.pdf]

nature research

Corresponding author(s): Ramiro Iglesias-Bartolome

Last updated by author(s): Feb 21, 2020

Reporting Summary

Nature Research wishes to improve the reproducibility of the work that we publish. This form provides structure for consistency and transparency in reporting. For further information on Nature Research policies, see Authors & Referees and the Editorial Policy Checklist.

Statistics

For all statistical analyses, confirm that the following items are present in the figure legend, table legend, main text, or Methods section.

n/a	Confirmed	
☐	☒	The exact sample size (*n*) for each experimental group/condition, given as a discrete number and unit of measurement
☐	☒	A statement on whether measurements were taken from distinct samples or whether the same sample was measured repeatedly
☐	☒	The statistical test(s) used AND whether they are one- or two-sided<br>*Only common tests should be described solely by name; describe more complex techniques in the Methods section.*
☐	☒	A description of all covariates tested
☐	☒	A description of any assumptions or corrections, such as tests of normality and adjustment for multiple comparisons
☐	☒	A full description of the statistical parameters including central tendency (e.g. means) or other basic estimates (e.g. regression coefficient) AND variation (e.g. standard deviation) or associated estimates of uncertainty (e.g. confidence intervals)
☐	☒	For null hypothesis testing, the test statistic (e.g. *F*, *t*, *r*) with confidence intervals, effect sizes, degrees of freedom and *P* value noted<br>*Give P values as exact values whenever suitable.*
☒	☐	For Bayesian analysis, information on the choice of priors and Markov chain Monte Carlo settings
☐	☒	For hierarchical and complex designs, identification of the appropriate level for tests and full reporting of outcomes
☒	☐	Estimates of effect sizes (e.g. Cohen's *d*, Pearson's *r*), indicating how they were calculated

Our web collection on statistics for biologists contains articles on many of the points above.

Software and code

Policy information about availability of computer code

Data collection	LASX software was used for image acquisition in Leica SP8 confocal microscope. BZX software was used in Keyence BZ-X700 microscope for automated image acquisition.
Data analysis	For RNAseq data reads were trimmed using Cutadapt 1.18; Trimmomatic 0.36; alignment to the human reference genome, hg19, was performed using STAR 2.6.1; gene counts, TMM, TPM and differential analysis was performed with Partek Flow and PARTEK Flow GSA algorith (Partek E/M, PartekFlow 8.0.19.0819). Gene Ontology (GO) terms were obtained with ToppGene (https://toppgene.cchmc.org/enrichment.jsp; v2018 and 2019). Analysis of over-represented conserved transcription factor binding sites was performed with oPOSSUM Single Site Analysis (SSA) V3.0 (http://opossum.cisreg.ca/oPOSSUM3/). Canonical pathways and upstream regulators analysis were generated with Ingenuity Pathway Analysis (IPA v49932394, Ingenuity Systems, www.ingenuity.com). GraphPad Prism v7 was used for statistical analysis. Quantification of expression in IF images was done with the BZX analysis software (v1.3, Keyence) equipped with hybrid cell count and macro cell count. Blot images were processed and quantified using ImageLab software v5.2.1 (Bio-Rad).

For manuscripts utilizing custom algorithms or software that are central to the research but not yet described in published literature, software must be made available to editors/reviewers. We strongly encourage code deposition in a community repository (e.g. GitHub). See the Nature Research guidelines for submitting code & software for further information.

Data

Policy information about availability of data

All manuscripts must include a data availability statement. This statement should provide the following information, where applicable:

- Accession codes, unique identifiers, or web links for publicly available datasets
- A list of figures that have associated raw data
- A description of any restrictions on data availability

RNAseq primary and processed data generated in this manuscript is available from GEO under accession numbers GSE137410, GSE137531 and GSE136876. Processed RNAseq data is provided in Supplementary Dataset 1, 2 and 3. The source data underlying Figs 1b, 1c, 1d, 1e, 1f, 2a, 2g, 2j, 2k, 2l, 3b, 3c, 3d, 3e, 3f, 3k, 4a,

4b, 4c, 4d, 4e, 4f, 5c, 5d, 5e, 5f, 6d, 6e, and Supplementary Figs 1a to f, 1g, 1h, 1i, 2a, 2b, 2c, 2f, 2g, 2i, 2j, 3a, 3b, 3c, 3d, are provided as a Source Data file.

Field-specific reporting

Please select the one below that is the best fit for your research. If you are not sure, read the appropriate sections before making your selection.

☒ Life sciences ☐ Behavioural & social sciences ☐ Ecological, evolutionary & environmental sciences

For a reference copy of the document with all sections, see nature.com/documents/nr-reporting-summary-flat.pdf

Life sciences study design

All studies must disclose on these points even when the disclosure is negative.

Sample size	No sample size calculation was performed. Sample sizes were chosen based on observed variability in preliminary assays and sample size was determined to be adequate based on the consistency of the results between replicates.
Data exclusions	No data points were excluded in the figures.
Replication	Replication numbers are indicated in the figure legends.
Randomization	No randomization was possible for animal experiments since mice were selected by genotype. All control animals were litermate controls, so no covariates were used during statistical analysis. For experiments involving cells, all control and treatment wells were plated at the same time and treatment was assigned to consecutive wells following visual inspection of homogeneous cell health across all wells. Multiplicity of infection, total amount of DNA or siRNA transfected, volume of transfection reagent, time of transfection or viral transduction, harvesting time, fixation time and/or treatment length were the same for control and treatment conditions, so no covariates were used during statistical analysis.
Blinding	Blinding was not performed during experiments and quantifications since same investigators were performing all procedures. Automated methods of data recording and analysis were adopted when possible to prevent user bias.

Reporting for specific materials, systems and methods

We require information from authors about some types of materials, experimental systems and methods used in many studies. Here, indicate whether each material, system or method listed is relevant to your study. If you are not sure if a list item applies to your research, read the appropriate section before selecting a response.

Materials & experimental systems

n/a	Involved in the study	
☐	☒	Antibodies
☐	☒	Eukaryotic cell lines
☒	☐	Palaeontology
☐	☒	Animals and other organisms
☒	☐	Human research participants
☒	☐	Clinical data

Methods

n/a	Involved in the study	
☒	☐	ChIP-seq
☒	☐	Flow cytometry
☒	☐	MRI-based neuroimaging

Antibodies

Antibodies used

Myc tag antibody (Cell Signaling; clone no. 71D10; catalogue no. 2278; 1:1000), Lot number: 5
anti GFP (Cell Signaling; clone no. D5.1; catalogue no. 2956; 1:2000), Lot number: 4
Cyclin D1 (Cell Signaling; clone no. 92G2; catalogue no. 2978; 1:1000), Lot number: 12
LATS1 (Cell Signaling; clone no. C66B5; catalogue no. 3477; 1:1000), Lot number: 7
anti-HA tag antibody (Cell Signaling; clone no. C29F4; catalogue no. 3724; 1:1000), Lot number: 3
E2F1 (Cell Signaling; catalogue no. 3742; 1:1000), Lot number: 4
anti-GAPDH (Cell Signaling; clone no. 14C10; catalogue no. 2118; 1:2000), Lot number: 7
Cleaved-PARP (Cell Signaling; clone no. D64E10; catalogue no. 5625; 1:1000). Lot number: 13
anti-FLAG tag antibody (Cell Signaling; clone no. 9A3; catalogue no. 8146; 1:1000), Lot number: 8
Total β-Catenin (Cell Signaling; clone no. D10A8; catalogue no. 8480; 1:1000), Lot number: 5
Phospho-Rb (Ser807/811) (Cell Signaling; clone no. D20B12; catalogue no. 8516; 1:100), Lot number: 6
Cleaved-Caspase3 (Cell Signaling; clone no. 5A1E; catalogue no. 9664; 1:1000), Lot number: 45
KLF4 (Cell Signaling; clone no. D1F2; catalogue no. 12173; 1:1000), Lot number: 2
PCNA (Cell Signaling; catalogue no. 13110S; 1:200), Lot number: 4
Pan-TEAD (Cell Signaling; clone no. D3F7L; catalogue no. 13295; 1:1000), Lot number: 2
YAP1 (Cell Signaling; clone no. D8H1X; catalogue no. 14074; 1:2000), Lot number: 4
CYR61 (Cell Signaling; clone no. D4H5D; catalogue no. 14479; 1:1000), Lot number: 1
Active β-Catenin (Cell Signaling; clone no. D2U8Y; catalogue no. 19807; 1:1000), Lot number: 1

Cyclin E1 (Cell Signaling; clone no. D7T3U; catalogue no. 20808; 1:1000), Lot number: 1
p63 (Cell Signaling; clone no. D9L7L; catalogue no. 39692; 1:1000), Lot number: 1
CD45 (Cell Signaling; clone no. D3F8Q; catalogue no. 70257; 1:100). Lot number: 1
anti-FLAG tag antibody (Biolegend; clone no. L5; catalogue no. 637304; 1:100), Lot number: B185584
anti-HA tag antibody (Covance; clone no. 16B12; catalogue no. MMS-101R; 1:100), Lot number: B220850
Loricrin (BioLegend; catalogue no. 905101; 1:100), Lot number: D14KF02261
KRT10 (BioLegend; catalogue no. 905401; 1:200-1:1000), Lot number: B274609
KRT5 (BioLegend; catalog no. 905501; 1:1000), Lot number: N/A
KRT5 (BioLegend; catalog no. 905901; 1:200), Lot number: B271562
GFP Polyclonal Antibody Alexa Fluor 488 (ThermoFisher; catalogue no. A-21311;1:500), Lot number: 1891008
GFP Polyclonal Antibody (ThermoFisher; catalogue no. A-6455; 1:500), Lot number: 2044346
mouse KLF4 (R&D; catalogue no. AF3158; 1:200), Lot number: WRR061804
p53 (DAKO; clone no. DO-7; catalogue no. GA616; 1:1000), Lot number: 20011629
Involucrin (SantaCruz; catalogue no. sc-21748; 1:500), Lot number: D1117
GAL4 (SantaCruz; catalogue no. SC510; 1:1000), Lot number: K2718
p16 (SantaCruz; catalogue no. sc-56330; 1:200), Lot number: I2512
TAZ (Cell Signaling; clone no. V386; catalogue no. 4883; 1:1000), Lot number: 3

Validation

Myc tag antibody (Cell Signaling; clone no. 71D10; catalogue no. 2278; 1:1000), , #Citations: 266 , Citations (CiteAb link): https://www.citeab.com/antibodies/123135-2278-myc-tag-71d10-rabbit-mab?des=4d7cdc11a678c83c , Validation from manufacturer related to application in our study: Validated and Approved for Western Blotting, Immunoprecipitation , Validation from our study: Gives band at expected molecular weight in western blot analysis .
anti GFP (Cell Signaling; clone no. D5.1; catalogue no. 2956; 1:2000), , #Citations: 210 , Citations (CiteAb link): https://www.citeab.com/antibodies/124367-2956-gfp-d5-1-xp-rabbit-mab?des=3095eeaa379e7187 , Validation from manufacturer related to application in our study: Validated and Approved for Western Blotting, Immunofluorescence (Immunocytochemistry), Immunohistochemistry (Paraffin) , Validation from our study: Gives band at expected molecular weight in western blot analysis .
Cyclin D1 (Cell Signaling; clone no. 92G2; catalogue no. 2978; 1:1000), , #Citations: 556 , Citations (CiteAb link): https://www.citeab.com/antibodies/124400-2978-cyclin-d1-92g2-rabbit-mab?des=79b5cbad229a3594 , Validation from manufacturer related to application in our study: Validated and Approved for Western Blotting , Validation from our study: Gives band at expected molecular weight in western blot analysis .
LATS1 (Cell Signaling; clone no. C66B5; catalogue no. 3477; 1:1000), , #Citations: 108 , Citations (CiteAb link): https://www.citeab.com/antibodies/122971-3477-lats1-c66b5-rabbit-mab?des=bfeb80be8dba6505 , Validation from manufacturer related to application in our study: Validated and Approved for Western Blotting , Validation from our study: Gives band at expected molecular weight in western blot analysis .
anti-HA tag antibody (Cell Signaling; clone no. C29F4; catalogue no. 3724; 1:1000), , #Citations: 642 , Citations (CiteAb link): https://www.citeab.com/antibodies/123379-3724-ha-tag-c29f4-rabbit-mab?des=92eabe9bc61dfdc0 , Validation from manufacturer related to application in our study: Validated and Approved for Western Blotting, Immunoprecipitation , Validation from our study: Gives band at expected molecular weight in western blot analysis .
E2F1 (Cell Signaling; catalogue no. 3742; 1:1000), , #Citations: 100 , Citations (CiteAb link): https://www.citeab.com/antibodies/123407-3742-e2f-1-antibody?des=0868a0db3ba0ca75 , Validation from manufacturer related to application in our study: Validated and Approved for Western Blotting , Validation from our study: Gives band at expected molecular weight in western blot analysis .
anti-GAPDH (Cell Signaling; clone no. 14C10; catalogue no. 2118; 1:2000), , #Citations: 1715 , Citations (CiteAb link): https://www.citeab.com/antibodies/125120-5174-gapdh-d16h11-xp-rabbit-mab?des=68f0a23d986c5e59 , Validation from manufacturer related to application in our study: Validated and Approved for Western Blotting , Validation from our study: Gives band at expected molecular weight in western blot analysis .
Cleaved-PARP (Cell Signaling; clone no. D64E10; catalogue no. 5625; 1:1000). , #Citations: 545 , Citations (CiteAb link): https://www.citeab.com/antibodies/125548-5625-cleaved-parp-asp214-d64e10-xp-rabbit-mab?des=c68643dfbd1fd7f6 , Validation from manufacturer related to application in our study: Validated and Approved for Western Blotting , Validation from our study: Gives band at expected molecular weight in western blot analysis under apoptotic but not control conditions .
anti-FLAG tag antibody (Cell Signaling; clone no. 9A3; catalogue no. 8146; 1:1000), , #Citations: 128 , Citations (CiteAb link): https://www.citeab.com/antibodies/125084-8146-dykddddk-tag-9a3-mouse-mab-binds-to-same-epit?des=d19233c4311e676d , Validation from manufacturer related to application in our study: Validated and Approved for Western Blotting, Immunoprecipitation , Validation from our study: Gives band at expected molecular weight in western blot analysis .
Total β-Catenin (Cell Signaling; clone no. D10A8; catalogue no. 8480; 1:1000), , #Citations: 502 , Citations (CiteAb link): https://www.citeab.com/antibodies/125282-8480-catenin-d10a8-xp-rabbit-mab?des=e96be5f96c3d4798 , Validation from manufacturer related to application in our study: Validated and Approved for Western Blotting , Validation from our study: Gives band at expected molecular weight in western blot analysis .
Phospho-Rb (Ser807/811) (Cell Signaling; clone no. D20B12; catalogue no. 8516; 1:100), , #Citations: 135 , Citations (CiteAb link): https://www.citeab.com/antibodies/125330-8516-phospho-rb-ser807-811-d20b12-xp-rabbit-mab?des=2aa1320432f1f515 , Validation from manufacturer related to application in our study: Validated and Approved for Immunofluorescence (Immunocytochemistry) , Validation from our study: Gives nuclear staining in Immunofluorescence .
Cleaved-Caspase3 (Cell Signaling; clone no. 5A1E; catalogue no. 9664; 1:1000), , #Citations: 4300 , Citations (CiteAb link): https://www.citeab.com/antibodies/126297-9661-cleaved-caspase-3-asp175-antibody?des=50b3c09def1880f9 , Validation from manufacturer related to application in our study: Validated and Approved for Western Blotting , Validation from our study: Gives band at expected molecular weight in western blot analysis under apoptotic but not control conditions .
KLF4 (Cell Signaling; clone no. D1F2; catalogue no. 12173; 1:1000), , #Citations: 16 , Citations (CiteAb link): https://www.citeab.com/antibodies/701405-12173-klf4-d1f2-rabbit-mab?des=4ee64379dc19a63f , Validation from manufacturer related to application in our study: Validated and Approved for Western Blotting and Immunoprecipitation , Validation from our study: Gives band at expected molecular weight in western blot analysis, this band is not present in siKLF4 cells, gives nuclear staining by Immunofluorescence .
PCNA (Cell Signaling; catalogue no. 13110S; 1:200), , #Citations: 149 , Citations (CiteAb link): https://www.citeab.com/antibodies/1539467-13110-pcna-d3h8p-xp-rabbit-mab?des=4b94af09a5f5c7eb , Validation from manufacturer related to application in our study: Validated and Approved for Immunohistochemistry (Paraffin), Immunofluorescence (Frozen),

Immunofluorescence (Immunocytochemistry) , Validation from our study: Gives nuclear staining in Immunofluorescence, in skin only labels basal proliferating cells (only basal cells proliferate in skin). .
Pan-TEAD (Cell Signaling; clone no. D3F7L; catalogue no. 13295; 1:1000), , #Citations: 18 , Citations (CiteAb link): https://www.citeab.com/antibodies/2043141-13295-pan-tead-d3f7l-rabbit-mab?des=b692e50ed647350f , Validation from manufacturer related to application in our study: Validated and Approved for Western Blotting and Immunoprecipitation , Validation from our study: Gives bands at expected molecular weights for TEADS in western blot analysis .
YAP1 (Cell Signaling; clone no. D8H1X; catalogue no. 14074; 1:2000), , #Citations: 149 , Citations (CiteAb link): https://www.citeab.com/antibodies/2397766-14074-yap-d8h1x-xp-rabbit-mab?des=8a7dfddb6cfa8650 , Validation from manufacturer related to application in our study: Validated and Approved for Western Blotting, Immunoprecipitation, Immunohistochemistry (Paraffin), Immunofluorescence (Immunocytochemistry) , Validation from our study: Gives band at expected molecular weight in western blot analysis, band diminishes in siYAP1 blots. .
CYR61 (Cell Signaling; clone no. D4H5D; catalogue no. 14479; 1:1000), , #Citations: 6 , Citations (CiteAb link): https://www.citeab.com/antibodies/2444863-14479-cyr61-d4h5d-xp-rabbit-mab?des=ba5d2d0e29b8ce16 , Validation from manufacturer related to application in our study: Validated and Approved for Western Blotting and Immunofluorescence (Immunocytochemistry) , Validation from our study: Gives band at expected molecular weight in western blot analysis, band diminishes in conditions were CYR61 mRNA decreases. .
Active β-Catenin (Cell Signaling; clone no. D2U8Y; catalogue no. 19807; 1:1000), , #Citations: 27 , Citations (CiteAb link): https://www.citeab.com/antibodies/3368883-19807-non-phospho-active-catenin-ser45-d2u8y?des=fe53df301e02ab5b , Validation from manufacturer related to application in our study: Validated and Approved for Western Blotting , Validation from our study: Gives band at expected molecular weight in western blot analysis .
Cyclin E1 (Cell Signaling; clone no. D7T3U; catalogue no. 20808; 1:1000), , #Citations: 34 , Citations (CiteAb link): https://www.citeab.com/antibodies/3394227-20808-cyclin-e1-d7t3u-rabbit-mab?des=2bacedc67f61b528 , Validation from manufacturer related to application in our study: Validated and Approved for Western Blotting , Validation from our study: Gives band at expected molecular weight in western blot analysis .
p63 (Cell Signaling; clone no. D9L7L; catalogue no. 39692; 1:1000), , #Citations: 0 , Citations (CiteAb link): https://www.citeab.com/antibodies/4165109-39692-p63-d9l7l-xp-rabbit-mab?des=56038400f09f935d , Validation from manufacturer related to application in our study: Validated and Approved for Western Blotting and Immunohistochemistry (Paraffin) , Validation from our study: Gives band at expected molecular weight in western blot analysis and nuclear localization in basal skin cells in Immunofluorescence .
CD45 (Cell Signaling; clone no. D3F8Q; catalogue no. 70257; 1:100). , #Citations: 0 , Citations (CiteAb link): https://www.citeab.com/antibodies/6115090-70257-cd45-d3f8q-rabbit-mab?des=31b6b04bb405e5ce , Validation from manufacturer related to application in our study: Validated and Approved for Immunofluorescence and Immunohistochemistry. , Validation from our study: Staining in Immunofluorescence in skin is primarly located were immune cells are expected (mainly dermal sites). .
anti-FLAG tag antibody (Biolegend; clone no. L5; catalogue no. 637304; 1:100), , #Citations: 9 , Citations (CiteAb link): https://www.citeab.com/antibodies/526009-637304-purified-anti-dykddddk-tag-antibody?des=c35f524341c93c5b , Validation from manufacturer related to application in our study: Each lot of this antibody is quality control tested by Western blotting. , Validation from our study: Gives band at expected molecular weight in western blot analysis .
anti-HA tag antibody (Covance; clone no. 16B12; catalogue no. MMS-101R; 1:100), , #Citations: 22 , Citations (CiteAb link): https://www.citeab.com/antibodies/2863941-901515-anti-ha-11-epitope-tag-antibody?des=eb1b8cea119db4de , Validation from manufacturer related to application in our study: Each lot of this antibody is quality control tested by Western blotting. , Validation from our study: Gives band at expected molecular weight in western blot analysis .
Loricrin (BioLegend; catalogue no. 905101; 1:100), , #Citations: 5 , Citations (CiteAb link): https://www.citeab.com/antibodies/2862383-905101-loricrin-polyclonal-antibody-purified?des=6f7e5abd2ac6357a , Validation from manufacturer related to application in our study: Each lot of this antibody is quality control tested by immunohistochemical staining. , Validation from our study: Staining in Immunofluorescence in skin is primarly located in highly differentiated suprabasal layers .
KRT10 (BioLegend; catalogue no. 905401; 1:200-1:1000), , #Citations: 9 , Citations (CiteAb link): https://www.citeab.com/antibodies/2862386-905401-keratin-10-polyclonal-antibody-purified?des=2ba08fb0ff947a4b , Validation from manufacturer related to application in our study: Each lot of this antibody is quality control tested by immunohistochemical staining. , Validation from our study: Gives band at expected molecular weight in western blot analysis, staining in Immunofluorescence in skin is primarly located in differentiated suprabasal layers .
KRT5 (BioLegend; catalog no. 905501; 1:1000), , #Citations: 45 , Citations (CiteAb link): https://www.citeab.com/antibodies/2862387-905501-keratin-5-polyclonal-antibody-purified?des=23b3102efbe2fe7e , Validation from manufacturer related to application in our study: Each lot of this antibody is quality control tested by immunohistochemical staining. , Validation from our study: Gives band at expected molecular weight in western blot analysis, staining in Immunofluorescence in skin is primarly located in basal layer .
KRT5 (BioLegend; catalog no. 905901; 1:200), , #Citations: 18 , Citations (CiteAb link): https://www.citeab.com/antibodies/2862391-905901-keratin-5-polyclonal-chicken-antibody-purifi?des=ddfc5debb4609bc9 , Validation from manufacturer related to application in our study: Each lot of this antibody is quality control tested by immunohistochemical staining. , Validation from our study: Gives band at expected molecular weight in western blot analysis, staining in Immunofluorescence in skin is primarly located in basal layer .
GFP Polyclonal Antibody Alexa Fluor 488 (ThermoFisher; catalogue no. A-21311;1:500), , #Citations: 287 , Citations (CiteAb link): https://www.citeab.com/antibodies/2401297-a-21311-gfp-polyclonal-antibody-alexa-fluor-488?des=b149f5d03770f462 , Validation from manufacturer related to application in our study: Tested by Microplate Assay, fold dilution, gainst GFP , Validation from our study: Gives band at expected molecular weight in western blot analysis .
GFP Polyclonal Antibody (ThermoFisher; catalogue no. A-6455; 1:500), , #Citations: 963 , Citations (CiteAb link): https://www.citeab.com/antibodies/2401379-a-6455-gfp-polyclonal-antibody?des=a8957a449494f768 , Validation from manufacturer related to application in our study: Tested by Microplate Assay, fold dilution, gainst GFP , Validation from our study: Gives band at expected molecular weight in western blot analysis .
mouse KLF4 (R&D; catalogue no. AF3158; 1:200), , #Citations: 74 , Citations (CiteAb link): https://www.citeab.com/antibodies/689842-af3158-mouse-klf4-antibody?des=23bb3e1631d281ef , Validation from manufacturer related to application in our study: Antigen Affinity-purified. Validated for Western Blot and Immunocytochemistry. , Validation from our study: Staining in Immunofluorescence in skin is primarly located in highly differentiated suprabasal layers .
p53 (DAKO; clone no. DO-7; catalogue no. GA616; 1:1000), , #Citations: 370 , Citations (CiteAb link): https://www.citeab.com/antibodies/2390686-m7001-p53-protein-concentrate?des=d4a38c7efbd6b6e5 , Validation from manufacturer related to

application in our study: SDS-PAGE analysis of immunoprecipitates formed between lysate of the BT474 breast cancer cell line and the antibody shows reaction with a 53 kDa protein corresponding to p53. In IHC, the antibody labels mutant-type p53 in the A431 cell line and wild-type p53 in the SVK14 cell line (SV40-transformed keratinocyte line) , Validation from our study: Gives band at expected molecular weight in western blot analysis .
Involucrin (SantaCruz; catalogue no. sc-21748; 1:500), , #Citations: 36 , Citations (CiteAb link): https://www.citeab.com/antibodies/801397-sc-21748-involucrin-antibody-sy5?des=b6bb83aebb2637c7 , Validation from manufacturer related to application in our study: Involucrin (SY5) is recommended for detection of involucrin of mouse, rat and human origin by Western Blotting, immunofluorescence and immunohistochemistry , Validation from our study: Gives band at expected molecular weight in western blot analysis .
GAL4 (SantaCruz; catalogue no. SC510; 1:1000), , #Citations: 310 , Citations (CiteAb link): https://www.citeab.com/antibodies/794778-sc-510-gal4-antibody-rk5c1?des=18121fa32ee284a8 , Validation from manufacturer related to application in our study: GAL4 (DBD) (RK5C1) is recommended for detection of GAL4 DNA binding domain by Western Blotting and immunoprecipitation , Validation from our study: Gives band at expected molecular weight in western blot analysis .
p16 (SantaCruz; catalogue no. sc-56330; 1:200), , #Citations: 74 , Citations (CiteAb link): https://www.citeab.com/antibodies/809810-sc-56330-p16-jc8?des=2b02fbb8527e20bd , Validation from manufacturer related to application in our study: p16 (JC8) is recommended for detection of p16 of human origin by WesternBlotting , Validation from our study: Gives band at expected molecular weight in western blot analysis .
TAZ (Cell Signaling; clone no. V386; catalogue no. 4883; 1:1000), , #Citations: 59 , Citations (CiteAb link): https://www.citeab.com/antibodies/124835-4883-taz-v386-antibody?des=3e1426d0af0cef88 , Validation from manufacturer related to application in our study: Validated and Approved for Western Blotting , Validation from our study: Gives band at expected molecular weight in western blot analysis, band diminishes in siTAZ blots. .

Eukaryotic cell lines

Policy information about cell lines

Cell line source(s)	HEK293 cells were obtained from AddexBio. Lenti-X™ 293T cells from Takara Bio. N/TERT-2G keratinocyte cell line was provided by Ellen H. van den Bogaard (Radboud University Medical Center, Nijmegen, The Netherlands) and James Rheinwald (Brigham and Women's Hospital, Boston, MA, USA)
Authentication	HEK293 cells and Lenti-X™ 293T were obtained directly from providing company and not further authenticated. N/TERT-2G cells were authenticated by STR profiling. Previous STR profile is not available for N/TERT-2G cells. Results from STR profile for N/TERT-2G cells were: TH01 8, 9.3; D5S818 11, 12; D13S317 8, 12; D7S820 9, 11; D16S539 10, 11; CSF1PO 10, 12; vWA 16, 18; TPOX 8, 11; Amelogenin X, Y.
Mycoplasma contamination	All cell lines tested negative for mycoplasma
Commonly misidentified lines (See ICLAC register)	No commonly misidentified cells were used.

Animals and other organisms

Policy information about studies involving animals; ARRIVE guidelines recommended for reporting animal research

Laboratory animals	Mus musculus. TRE-TEADi transgenic mice were generated in C57 background. K5-rtTA mice were FVB/N. Both male and female mice were used in the studies and all experiments were conducted using littermate controls. Treatment was started between weeks 6 to 10 after birth.
Wild animals	This study did not involve wild animals.
Field-collected samples	This study did not involve field-collected samples.
Ethics oversight	All animal studies were carried out according to approved protocols from the NIH-Intramural Animal Care and Use Committee (ACUC) of the National Cancer Institute, in compliance with the Guide for the Care and Use of Laboratory Animals.

Note that full information on the approval of the study protocol must also be provided in the manuscript.
