# Supplementary material for: YAP1/TAZ-TEAD transcriptional networks maintain skin homeostasis by regulating cell proliferation and limiting KLF4 activity
Source: Nat Commun. 2020 Mar 19;11:1472. doi: 10.1038/s41467-020-15301-0 (PMC7081327; doi:10.1038/s41467-020-15301-0)
Supplement: Supplementary file 7 — Description of Additional Supplementary Files [file 41467_2020_15301_MOESM7_ESM.pdf]

Title: Supplementary Data 1:
Description: Differential expression analysis using PARTEK Flow GSA algorithm in N/TERT2G keratinocytes transduced with GFP (Ad-GFP, control) or TEADi (Ad-TEADi) for 12, 24 or 48hs. For each time point, triplicate samples were used for Ad-GFP and Ad-TEADi.

Title: Supplementary Data 2:
Description: Differential expression analysis using PARTEK Flow GSA algorithm in N/TERT2G keratinocytes transduced with siCon or siYAP1/TAZ for 48hs. siCon n=3, siYAP1/TAZ n=4.

Title: Supplementary Data 3:
Description: Differential expression analysis using PARTEK Flow GSA algorithm in N/TERT2G keratinocytes transduced with siCon or siKLF4 for 48hs. siCon n=3, siKLF4 n=4. We hope the revised manuscript answers all the concerns and you and the Reviewer find it appropriate for publication. Thank you for your consideration.
